# Complete testing coverage for the early infant diagnosis algorithm and associated factors among infants exposed to HIV, Uganda, 2017–2019

**DOI:** 10.1371/journal.pone.0324338

**Published:** 2025-06-10

**Authors:** Rebecca Akunzirwe, Julie R. Harris, Peter Chris Kawungezi, Mercy W. Wanyana, Tom Lutalo, Phoebe Monalisa Namukanja, Augustina Delaney, Richard Migisha, Esther Nyamugisa, Doreen Ondo, Philip Kasibante, Daniel Kadobera, Lilian Bulage, Jane Frances Zalwango, Alex Riolexus Ario, Linda Kisaakye Nabitaka

**Affiliations:** 1 Uganda Public Health Fellowship Program, Uganda National Institute of Public Health, Kampala, Uganda; 2 Division of Global Health Protection, US Centers for Disease Control and Prevention, Kampala, Uganda; 3 Rakai Health Sciences Program, Kalisizo, Uganda; 4 Division of Global HIV and Tuberculosis, US Centers for Disease Control and Prevention, Kampala, Uganda; 5 United Nations Children’s Fund, Kampala, Uganda; 6 AIDS Control Program, Ministry of Health, Kampala, Uganda; University of Zimbabwe Faculty of Medicine: University of Zimbabwe College of Health Sciences, ZIMBABWE

## Abstract

**Background:**

Early infant diagnosis (EID) facilitates early initiation into HIV care for identified HIV-positive infants. According to the Uganda Ministry of Health, EID testing algorithm, testing for infants exposed to HIV (IEH) should occur at <6 weeks, 9 and 18 months of age, and 6 weeks after stopping breastfeeding. Uganda has faced challenges with loss to follow-up (LTFU) of IEH for EID. We assessed complete testing coverage (CTC) to the EID algorithm for IEH and associated factors.

**Methods:**

We analyzed data from the ‘Impact of the National Program for the Prevention of Vertical Transmission (PVT) of HIV in Uganda (2017−2019)’ study. Mothers living with HIV whose infants tested HIV-negative at 4–12 weeks were enrolled in a prospective cohort (2017 − 2018) and followed until the IEH tested positive, died, was LTFU, or reached 18 months of age. We computed the proportion of IEH tested according to the EID algorithm among surviving infants. CTC was defined as undergoing HIV tests at three designated time points (excluding the 6 weeks after breastfeeding cessation) if HIV negative. IEH who were diagnosed with HIV but were tested at all recommended tests until that point were also considered to have CTC. We evaluated factors associated with CTC using modified Poisson regression.

**Results:**

Among 1,804 IEH, 912 (51%) were male. Of the 1,804 IEH at baseline, 27 (1%) died. Among the 1,777 IEH included in the primary outcome analysis, 1,282 (72%) completed the study and 941 (53%) infants had CTC according to the EID testing algorithm including 40 (2%) who tested HIV-positive. Perceived discrimination due to HIV status [RR = 0.77, 95%CI (0.65–0.92)], having fewer pregnancies [RR = 0.97, 95%CI (0.68–0.99)], and reporting sexual violence [RR = 0.82, 95%CI (0.73–0.93)] by the mother of IEH were associated with non-CTC.

**Conclusion:**

About half of IEH were tested at the recommended time points. Interventions to address stigma and sexual violence for mothers may improve CTC for the EID algorithm. Investigations are needed to explore associations between sexual violence, parity, and CTC for the EID algorithm.

## Introduction

In 2023, there were approximately 120,000 incident HIV infections among children less than 15 years old globally [1]. In 2022, Sub-Saharan Africa accounted for 84% of these infections; Uganda accounted for approximately 5% [2,3]. While progress has been made towards achieving the Joint United Nations Program on HIV/AIDS (UNAIDS) 95-95-95 targets, advancements for children living with HIV (CLHIV) have been comparatively slower on a global scale [4]. For instance, in 2023, 34% of CLHIV less than 15 years did not know their status and about 43% of CLHIV were not accessing anti-retroviral therapy (ART) [1].

Mortality among children living with HIV under 2 years can be rapid unless they are started on ART, which may be delayed if caregivers are unaware of a child’s HIV status [5–7]. To reduce this mortality, the Ministry of Health (MoH), Uganda recommends an early infant diagnosis (EID) strategy through which infants exposed to HIV (IEH) are tested by Deoxyribonucleic Acid Polymerase Chain Reaction (DNA PCR) within the first six weeks of life (1^st^ PCR) to guide decision-making about care and treatment [8]. In addition, DNA PCR should be done at 9 months (2^nd^ PCR), 6 weeks after cessation of breastfeeding (3^rd^ PCR), and finally, serology at 18 months (rapid test) if DNA PCR tests are negative. If the infant is found to be HIV-positive at any stage, they are linked to care and treatment [8]. The Uganda MoH implemented the 1st and 3rd PCR and serology tests for IEH from 2006 to 2010, followed by the 2^nd^ PCR test in 2017 [9,10].

Similar to other low-income and high-HIV-burden countries, Uganda has faced challenges in the uptake and implementation of EID since its roll-out by MoH in 2006 [11–14]. In 2020, only 61% of children less than 10 years living with HIV had a known HIV status [15]. In a 2014 evaluation of EID at 12 health facilities in Uganda, 52% of IEH had a DNA PCR test by 2 months of age, and 55% had a DNA PCR test after finishing breastfeeding [9]. In 2017, the Uganda MoH developed and implemented several interventions to enhance the retention and follow-up of mother-baby pairs and strengthen the prevention of vertical transmission of HIV (PVT) [16], including tracking appointments of pregnant women and lactating mothers living with HIV, using expected dates of delivery to set up pre-appointments, bringing back mother-baby pair initiative, and birth cohort monitoring to ensure IEH adhered to EID [16].

Uganda made significant strides in the implementation of PVT interventions between 2006 and 2017 [9,17]. However, complete testing coverage (CTC) according to the EID testing algorithm has not been evaluated since the roll-out of interventions to enhance retention. We determined CTC according to the EID testing algorithm and associated factors among IEH in Uganda, 2017─2019 to inform HIV control and prevention interventions.

## Methods

### Study design and data source

We utilized data from the ‘Impact of the National Program for Prevention of Vertical Transmission of HIV in Uganda’ study [18]. Mothers of childbearing age (15–49 years) and their infants were enrolled from immunisation and postnatal clinics of 206 health facilities from 2017 to 2018 (baseline phase of the study). The 206 facilities, comprising 152 national PVT (non-DREAMS sites) and 54 DREAMS, were chosen for national representativeness [18]. DREAMS (Determined, Resilient, Empowered, AIDS-free, Mentored and Safe), a program targeting HIV reduction in adolescent girls and young women, operated in 10 districts in Uganda as of 2017. Selection of facilities involved stratified random sampling from low-, medium-, and high-volume facilities and simple random sampling to select mother-baby pairs within each facility [18]. IEH with a negative DNA PCR test at baseline (i.e., a negative test at 4–12 weeks old) and whose caregiver provided written informed consent to be followed up were included in this analysis. In the larger national impact evaluation study, the first follow-up visit was conducted when the infant was 6 months old and then after every 3 months until the infant turned 18 months old. In this analysis, we obtained information on infant HIV-status, whether alive or dead and whether they were returned for the HIV test at the EID testing time points. Infants who died were excluded from the analysis because we could not ascertain whether they would or would not have CTC if they had remained alive. Infants who tested positive are considered as having CTC according to the testing algorithm if they had had the recommended HIV tests until the time point they turned positive. Infants are defined as ‘lost to testing’ if they were not returned for an EID test at the specified time point (9 month and 18-month tests) with a window period of 6 weeks before or after the subsequent timepoint; they are regarded as having experienced the outcome of interest (non-CTC), even if they returned to care for another test. However, these infants are included in the denominator for testing coverage calculations. IEH who had a positive PCR results had a Dry Blood Spot for confirmatory DNA PCR HIV testing according to the MoH guidelines [17]. The follow up period extended until 2019.

### Study variables and data abstraction

The outcome variable in our study was CTC according to the EID algorithm, defined as being up to date with HIV testing for the infant at each stage (DNA PCR test within 4–6 weeks after birth, 9 months, and serology at 18 months with receipt of results at each stage). For the baseline test (6-week test), testing with a grace period of up to 12 weeks was allowed, with testing not permitted before 4 weeks. For the 9-month and 18-month tests, we considered a testing window of 6 weeks before and 6 weeks after the recommended time point. We abstracted data from the ‘Impact of the National Program for Prevention of Vertical Transmission of HIV in Uganda’ database on infant sex, the number of HIV tests done for each infant, and at what age the HIV test(s) was done. We also abstracted data on maternal exposures including age, marital status, ART status (whether or not they were on ART), lifetime experience of any violence (physical, sexual or emotional) from any perpetrator, stigma, HIV disclosure status to partner, time of ART initiation, place of infant delivery, facility level of receipt of care, number of antenatal care visits, gestational age at first antenatal care (ANC) visit, and alcohol use. Stigma was measured using the Stigma, Rights, and Vulnerabilities in HIV/AIDS research (STRIVE) measurement tool for people living with HIV in the parent study [19]. Participants were asked four questions about their experiences in the past year: (a) Has someone spoken negatively about you because of your HIV status? (b) Has someone verbally insulted, harassed, or threatened you due to your HIV status? (c) Have you been excluded from family activities because of your HIV status? (d) Have you been excluded from religious activities or places of worship due to your HIV status? [19].

Participants were categorised as ‘having experienced stigma’ if they answered ‘yes’ to any question and as ‘not experienced stigma’ if they answered ‘no’ to all questions.Alcohol use was measured according to the National Institute on Alcohol Abuse and Alcoholism questionnaire in the parent study [20]. We categorized women as: a) ‘at risk for alcohol-related problems’ if they had more than 7 drinks per week or more than 3 drinks per occasion, b) ‘heavy drinkers’ if they drank more than 14 drinks per week or more than 4 drinks per occasion, c) ‘safe’ if they drank at most 7 drinks per week or at most 3 drinks per occasion and d) ‘doesn’t drink’ if they did not consume alcohol at all for at least a year [20].

### Data management and statistical analysis

We exported data to Stata version 16.0 for analysis. Categorical variables were summarised as frequencies and percentages and presented in form of contingency tables. Numerical characteristics were summarised as medians and interquartile ranges since the data were non-normally distributed data. In determining the proportion of infants who had CTC according to the EID algorithm and assessing associated factors, we excluded infants who died from the analysis between time points. We categorized testing coverage according to the EID algorithm as ‘complete’ vs ‘non-complete. Infants were categorized has having CTC if the infant underwent three recommended HIV tests at the three specified time points (DNA PCR test within 4 to 6 weeks after birth, 9 months, and serology at 18 months with receipt of results at each stage). For the baseline test (6-week test), testing was recommended between 4 to 6 weeks after birth, but a grace period of up to 12 weeks was allowed, with testing not permitted before 4 weeks. For the 9-month and 18-month tests, we considered a testing window of 6 weeks before and 6 weeks after the recommended time point. Infants who were diagnosed with HIV at any stage but had the recommended HIV tests until the time point they turned positive were considered as having CTC. We classified testing as ‘non-complete’ if these conditions were not met. To assess predictors of CTC according to the EID algorithm, we employed a modified Poisson regression analysis. We used clustered robust standard errors to correct for overestimating the standard errors and account for clustering by site. The measure of association was the risk ratio, because the outcome was not rare and this was a cohort analysis and risk could be directly estimated from the analysis [21–23]. Risk ratios between individual predictors and CTC according to the EID algorithm at the bivariable level were assessed, and those with a p-value less than 0.2 were considered for the multivariable analysis. We assessed for interaction using the likelihood ratio test by comparing full and reduced models. We did not find any statistical interaction between the variables. We assessed for confounding variables by comparing risk ratios for CTC according to the EID algorithm of unadjusted model and adjusted models. Variables that caused a difference between the risk ratios of both models of at least 10% were considered confounders, but no confounding was identified. Variables with a p-value less than 0.05 were regarded as statistically significant. We performed a sensitivity analysis by examining the outcomes if the infants who had died were categorized as CTC or non-CTC (S1 Table).

### Ethical approval

This study was reviewed and approved by the Uganda Virus Research Institute (UVRI) Research Ethics Committee (UVRI-REC Federal Wide Assurance (FWA) No. 00001354) and the Uganda National Council for Science and Technology (FWA No. 00001293) under 45 C.F.R. part 46.101(c); 21 C.F.R. part 56. We were given permission by the MoH AIDS Control Program to utilize the survey data. Makerere University School of Public Health gave approvals to the secondary data analysis study. For this analysis, we received de-identified data on 4^th^ February, 2023 to protect the confidentiality of the participants.

## Results

### Characteristics of the study population

At baseline (2017 − 2018), there were 1,804 IEH enrolled. Of these, 912 (51%) were male and 530 (29%) were less than six weeks old. Regarding the mothers of these infants, 1,217 (67%) were 25 years old or older, while 111 (6%) were aged between 15 and 19 years. Additionally, 1,299 (72%) reported being married, 199 (11%) were cohabiting, and 198 (11%) reported never having been married; 397 (22%) received care from a health center IV or hospital. A total of 228 (13%) reported ever experiencing sexual violence from any perpetrator, 195 (11%) reported being discriminated against due to HIV status and 235 (13%) had disclosed their HIV status (Table 1).

**Table 1:** Baseline characteristics of 1,804 mothers living with HIV and their exposed infants, Uganda, 2017 − 2018.

Characteristic	n	(%)
**Infant characteristics**		
Male sex (vs female) (n = 1793) ^¥^	912	(51%)
Age > 6 weeks (vs ≤ 6) (n = 1794) ^¥^	1264	(70%)
**Maternal characteristics**		
Age (years) (n = 1785) ^¥^		
15-19	111	(6%)
20-24	457	(25%)
25+	1217	(67%)
Marital status (n = 1696) ^¥^		
Never married	198	(11%)
Married	1299	(72%)
Cohabiting	199	(11%)
Received health care at Health center II, III, or clinic level (vs Health center IV or hospital)	1407	(78%)
Number of pregnancies (n = 1725) ^¥^		
Median (min, max)	3 (1,14)	
1	206	(11%)
2-4	980	(54%)
5+	539	(30%)
Gestational age at first ANC (n = 1620) ^¥^		
1st trimester	544	(30%)
2nd trimester	963	(53%)
3rd trimester	113	(6%)
More than 4 ANC visits (vs < 4) (n = 1611) ^¥^	1005	(56%)
Where infant was born (n = 1751) ^¥^		
Public facility	1198	(66%)
Private clinic	357	(20%)
Home	196	(11%)
ART initiation (n = 1485) ^¥^		
Before pregnancy	980	(54%)
During pregnancy	438	(24%)
After Delivery	45	(2%)
Not on ART	22	(1%)
Disclosure of HIV status, no (vs yes) ^(^n = 1493) ^¥^	1258	(70%)
Reported experience of sexual violence, no (vs yes)	1576	(87%)
Reported experience of physical violence, no (vs yes)	1262	(70%)
Felt discriminated against due to HIV status, no (vs yes) (n = 1459) ^¥^	1264	(70%)
Alcohol consumption (n = 1637) ^¥^		
Heavy drinkers/ at risk for alcohol-related problems	53	(3%)
Safe	133	(7%)
Does not drink	1451	(80%)

¥ variable has missing values (some participants did not respond to the question). Records with missing information are not shown in the table. For the variable, alcohol consumption, we combined ‘heavy drinker’ and ‘at risk for alcohol-related problems’ due to the small sample size in each category.

### Complete testing coverage according to the early infant diagnosis algorithm among infants exposed to HIV

Of the 1,804 IEH at baseline, 27 (1%) died and 40 (2%) turned HIV-positive; 1,282 (72%) of the IEH who were HIV-negative and did not die completed the study (Table 2). 941/1,777 (53%) infants who did not die by age 18 months, had CTC according to the EID testing algorithm.

**Table 2:** Early infant diagnosis cascade for 1,804 Infants exposed to HIV, Uganda, 2017-2019.

Time point	Number eligible for testing (n)	Died (n)	eligible for testing who died (%)	Number tested (n)		Missing results (n)	Missing among the tested (%)	Were not returned for testing at given recommended time point (n)	eligible for testing who didn’t return for testing at given time point (%)	Positive	positive among the tested (%)	Negative (n)	negative among tested (%)	Number with CTC at time point (n)	Number with CTC to date^a^ (n)	Total number of IEH at timepoint excluding deaths^b^ (n)	CTC to date among total infants at timepoint excluding deaths^c^ (%)
Baseline	1804	–	–	1804	162		9.0	–	–	37	2.1%	1605	89.0%	1642	1642	1804	91.0%
																
9months	1605	18	1.1%	1258	45		3.6%	329	20.5%	1	0.1%	1212	96.3%	1213	1250	1587	78.8%
																
18months	1212	9	0.7%	942	39		4.1%	261	21.5%	2	0.2%	901	95.6%	903	941	1203	78.2%
																
Total over 18 months	1804	27	1.5	1187^d^	246		20.7	590	32.7	40	3.4	901	75.9	903	941	1777	53.0

^a^ *-Number with CTC to date includes the number of IEH tested at that time point and the cumulative number of HIV positive until that testing point,*

^b^ *-The total number of infants excludes deaths,*

^c^ *-Percent of who had CTC to date among the total number of IEH excludes those who died,*

^d^ *-The total number tested over 18 months includes those who tested positive, negative, and those with missing results.*

At baseline (testing at 4–12 weeks), 1,605 (89%) were HIV-negative, 37 (2%) were HIV-positive, and 162 (9%) did not have their results returned from the laboratory. Among baseline-negative IEH, 1,212 (76%) were negative, 1 (0.06%) was positive, 18 (1%) died, and 329 (20%) were not tested and 45 (3%) did not have their results returned from the laboratory at 9 months. Among those negative at 9 months, 901 (74%) were negative at 18 months, 2(0.2%) were HIV-positive, 9 (0.7%) died, and 261 (22%) were not tested and 39 (3%) were tested but did not have their results returned from the laboratory (Table 2).

Overall, 10% (164/1,605) HIV-negative IEH at baseline were only tested at baseline; 26% (455/1,777) of those who were not known to have died were lost from care during the follow-up period (including the 164 tested only at baseline and 291 tested only at baseline and 9 months). 21% (381/1,777) returned for later HIV tests in the cascade (40 (10%) returned for the 9 months test and 360 (68%) returned for the 18 months test.

### Factors associated with complete testing coverage according to the early infant diagnosis algorithm among infants exposed to HIV

In bivariable modified Poisson regression analyses, we found partnered status, feeling discriminated against due to HIV status, having had fewer pregnancies, and reporting sexual violence reduced CTC according to the EID algorithm (Table 3). In multivariable analysis, reporting experience of discrimination [aRR = 0.77, 95%CI (0.65–0.92)], and reporting sexual violence [aRR = 0.82, 95%CI (0.73–0.93)] at baseline were less likely to have CTC according to the EID algorithm while those with more pregnancies [aRR = 1.03, 95%CI (1.01–1.46] were more likely to have CTC according to the EID algorithm (Table 3).

**Table 3:** Factors associated with complete testing coverage according to the early infant diagnosis algorithm among Infants exposed to HIV, Uganda, 2017-2019.

Characteristic	Categories	CTC as per EID (%)		cRR	(95% CI)	aRRaRR	(95% CI)
**Maternal characteristics**							
**Partnered status**	Have partner	99/193	(51%)	Ref		Ref	
	No partner	795/1473	(54%)	0.75**	(0.89-1.19)	0.98	(0.84-1.16)
**Facility level**	HCII	396/735	(54%)	Ref		Ref	
	HCIII	340/647	(53%)	1.00	(0.91-1.11)	1.03	(0.92-1.15)
	Hospital/HCIV	203/390	(52%)	0.91*	(0.81-1.03)	0.97	(0.84-1.12)
**Number of pregnancies, mean(±SD)**	–	4 (±2)		1.02**	(1.00-1.05)	1.03**	(1.01-1.46)
**Trimester of pregnancy at first ANC**	1^st^	287/531	(54%)	1.03	(0.93-1.14)		
	2^nd^	505/951	(53%)	Ref			
	3^rd^	61/112	(54%)	1.11	(0.93-1.32)		
**Alcohol consumption**	No Drink	745/1423	(52%)	Ref		Ref	
	Safe	81/131	(62%)	1.10*	(0.95-1.29)	1.06	(0.82-1.13)
	Heavy/at risk	27/53	(51%)	0.98	(0.74-1.27)	0.88	(0.64-1.21)
**When ART was initiated for mother**	Before pregnancy	513/966	(53%)	Ref			
	During pregnancy	227/428	(53%)	1.01	(0.91-1.12)		
	After delivery	20/44	(45%)	0.99	(0.74-1.32)		
**Reported experience of sexual violence**	No	802/1546	(52%)	Ref		Ref	
	Yes	137/226	(61%)	0.84**	(0.76-0.94)	0.82**	(0.73-0.93)
**Reported experience of physical violence**	No	774/1254	(62%)	Ref			
	Yes	74/119	(62%)	0.97	(0.83-1.13)		
**Disclosed HIV status**	No	658/1240	(53%)	Ref		Ref	
	Yes	114/228	(50%)	0.94	(0.82-1.08)	0.94	(0.82-1.09)
**Experienced discrimination**	No	675/1240	(54%)	Ref		Ref	
	Yes	82/194	(42%)	0.78**	(0.64-0.91)	0.77**	(0.65-0.92)
**Infant characteristics**							
**Place of birth for infant**	Public Facility	618/1180	(52%)	Ref		Ref	
	Private Clinic	190/348	(55%)	1.04	(0.93-1.16)	1.04	(0.91-1.18)
	Home	106/192	(55%)	1.06*	(0.93-1.22)	1.08	(0.92-1.27)

N for Complete testing coverage (CTC) according to the EID algorithm may not add up to 941 because of missingness for all variables, *p < 0.2, **p < 0.05. ‘Ref’ refers to the reference group. cRR-Crude Risk Ratio, aRR- Adjusted Risk Ratio

## Discussion

EID is important for reducing the impact of HIV, and CTC according to the EID algorithm is the first step in ensuring that infants are diagnosed and treated if they have HIV. During 2017–2019, about half of IEH in Uganda had CTC according to the EID algorithm and a quarter were lost from care. CTC was lower among infants of women who had fewer pregnancies, reported experiencing sexual violence, and who felt discriminated against because of their HIV status.

In this study, between one fifth of IEH missed HIV tests at the different time points. Of the infants who missed the testing at the different recommended time points, 26% were lost from the EID algorithm while 21% returned later than recommended. There was high loss from the EID testing at the 2^nd^ PCR (9-month test) and serology EID tests (18-month test), in which about 1 in 5 eligible IEH who did not die at the given timepoint did not re-test. The high loss of IEH at the 9-month timepoint may be due to the relatively recent introduction of this time point for testing (in July 2017 [10]. High losses of IEH at the 18-month time point have been reported in India where 40% of eligible IEH received the 18-months test [24] compared to 64% of eligible IEH who received a PCR test at 6 months after cessation of breastfeeding [25]. Poor coverage to the 18-month test has been linked to inadequate knowledge of the importance of completion of EID tests [26,27]. Previous studies have demonstrated that caregivers have also been less likely to bring an infant who has tested HIV-negative back for the last test unless they become ill [27]. Despite this, 21% of HEI who missed an HIV test returned to care, with 68% of those who returned to care returning for the 18-month test. This increased return rate is likely attributed to intensified efforts, such as active tracing and follow-up, as well as the availability of outreach clinics at this crucial timepoint The 18-month visit serves as the final outcome assessment to determine the HIV status of IEH, making it a pivotal moment for intervention and care [8].

Our results show an improvement since 2012 in CTC according to the EID algorithm, when >60% of infants who were eligible for a re-test did not do so at 6 weeks after cessation of breastfeeding, and 70% of those eligible for the rapid test at 18 months did not re-test [11]. This improvement could be attributable to interventions implemented by the Uganda MoH in 2017 that were aimed at strengthening EID services [9,16]. These initiatives include tracking appointments of pregnant women and lactating mothers living with HIV, using expected dates of delivery to set up pre-appointments, bringing back mother-baby pair initiative, and birth cohort monitoring [9,16]. A cluster-randomized trial at two government hospitals in Kenya in 2017 showed that infant tracking initiatives improved rates of testing within six weeks of life [28]. A systematic review of ten studies in low- and middle-income countries from 1990 to 2015 showed that tracking appointments of pregnant women and lactating mothers living with HIV improved retention of mothers living with HIV and their infants in PVT programs, including EID [29].

Mothers who reported feelings of being discriminated against by community members because of their HIV status were 23% less likely to have CTC according to the EID algorithm. Individuals who feel discriminated against because of their HIV status may be less likely to seek or be offered social support, because they anticipate rejection due to their HIV status [30,31]. Internalized stigma can lead to maladaptive health behaviors such as avoiding taking HIV medications [32]; this may similarly be associated with challenges with taking an infant for HIV diagnostic tests. Our study adds to the existing evidence from several low-income countries showing an association between feelings of discrimination due to HIV status and poor adherence to HIV-related services among persons living with HIV [33–35]. Addressing discrimination is essential to improving adherence to HIV-related services including CTC according to the EID testing algorithm.

Mothers who reported ever experiencing sexual violence were 18% less likely to have CTC according to the algorithm for their infants compared to those who did not report this experience. Available evidence indicates that women who have faced violence might refrain from disclosing their HIV status to partners, fearing potential intimate partner violence. This lack of disclosure can result in suboptimal adherence to PVT measures including EID, as it may inadvertently reveal the woman’s HIV status to her partner [36–38]. Additionally, studies propose that violence against women living with HIV worsens their mental health states and can cause depression, anxiety, and post-traumatic stress disorder, which are linked to poor adherence to HIV services [39–42]. Our results are similar to those of a cross-sectional study in Zambia in 2014 whereby women living with HIV who had experienced sexual violence had 74% lower odds of adherence to the PVT services in the postpartum period compared to those who hadn’t experienced sexual violence. Research aimed at closing the gap between policies addressing sexual violence against women and their actual implementation [43] may mitigate sexual violence and lead to greater CTC according to the EID testing algorithm.

Each additional pregnancy was associated with a 3% increase in CTC according to the EID diagnosis algorithm. Knowledge of PVT and EID was shown in one study to increase with increasing number of pregnancies; this may be due to a higher number of ANC visits during which the mother can become exposed to this information [44]. Our results are similar to those of a cross-sectional study in Ethiopia in 2019 where the odds of adhering to the PVT cascade increased by 5% for every increase in the number of pregnancies [45]. Greater attention to health education given to mothers with fewer pregnancies may improve CTC according to the EID algorithm.

This analysis had some limitations. First, children were included in the study only if they received the first prescribed HIV-PCR test at 6 weeks. As a result, children who missed the first test were excluded from later testing denominators. Due to the lack of data on IEH who did not receive the initial PCR test, we are unable to determine the true testing coverage for all eligible children at 6 weeks. However, our findings provide insight into CTC among IEH who completed the first HIV-PCR test and were followed up over time. Second, our study excludes the 6-weeks post-breastfeeding HIV test due to the lack of data on individual breastfeeding duration. As such, this study does not assess the full EID testing coverage but rather assess coverage for three timepoints (6 weeks, 9 months and 18 months test. Third, experience of violence and reported alcohol consumption are likely to be underreported due to social desirability which could bias the results toward the null. In addition, we assessed for lifetime experience of violence, which may be subject to recall bias; this could have attenuated the association between violence and CTC according to the EID testing algorithm. Fourth, there may be residual confounding from unmeasured variables, such as distance to the facility where the mother was getting HIV-related care for the baby or socioeconomic status. Fifth, some exposure variables had missing data; we could not determine if mothers with missing data were different from mothers without missing data in responses for a given variable. However, the proportion of mothers with missing responses for any variable was less than 20%, and we believe this had minimal impact on the validity of our study. For infants exposed to HIV lost to follow-up, there is uncertainty about whether this occurred because they sought care at another facility, potentially leading to misclassification as non-CTC according to the EID testing algorithm. Finally, we lack certainty regarding whether IEH who dropped out of the EID testing cascade either passed away or tested positive. There is a possibility that this group differed from IEH who tested negative at the 18-month HIV test.

## Conclusion

Fewer than half of IEH were had CTC according to the EID testing algorithm in Uganda during 2017–2019. Feelings of discrimination due to HIV status, reports of sexual violence, and fewer pregnancies were associated with lower CTC. Scale-up of PVT interventions, such as appointment tracking systems, could reduce the loss of mother-baby pairs in PVT programming, especially for women with fewer pregnancies and those who have experienced sexual violence. Investing in research on bridging the gap between the policy on sexual violence and the actual implementation of measures to prevent and address sexual violence could improve CTC according to the EID testing algorithm.

## Supporting information

S1 Table Sensitivity analysis for factors associated with complete testing coverage according to the early infant diagnosis algorithm among Infants exposed to HIV, Uganda, 2017–2019. (DOCX)

## References

[1] World Health Organisation. HIV statistics, globallyglobally and by WHO region, 2024; 2024 [cited 2025 Mar 4]. Available from: https://cdn.who.int/media/docs/default-source/hq-hiv-hepatitis-and-stis-library/j0482-who-ias-hiv-statistics_aw-1_final_ys.pdf?sfvrsn=61d39578_3

[2] UNAIDS. HIV and children; 2023 [cited 2025 Mar 4]. Available from: https://thepath.unaids.org/wp-content/themes/unaids2023/assets/files/thematic_fs_hiv_children.pdf

[3] Uganda AIDS Commission. Facts on HIV and AIDS in Uganda 2023 (Based on data ending 31st December 2022); 2023 [cited 2025 Mar 4]. Available from: https://www.uac.go.ug/media/attachments/2024/01/23/hiv-aids-factsheet-2023.pdf

[4] UNAIDS. The path that ends AIDS; 2023 [cited 2023 Sep 6]. Available from: https://www.unaids.org/en/resources/presscentre/pressreleaseandstatementarchive/2023/july/unaids-global-aids-update

[5] Mugavero MJ, Pence BW, Whetten K, Leserman J, Swartz M, Stangl D, et al. Predictors of AIDS-related morbidity and mortality in a southern U.S. Cohort. AIDS Patient Care STDS. 2007;21(9):681–90. doi: 10.1089/apc.2006.0167 17919095

[6] Bong C-N, Yu JK-L, Chiang H-C, Huang W-L, Hsieh T-C, Schouten EJ, et al. Risk factors for early mortality in children on adult fixed-dose combination antiretroviral treatment in a central hospital in Malawi. AIDS. 2007;21(13):1805–10. doi: 10.1097/QAD.0b013e3282c3a9e4 17690580

[7] Weldemariam SA, Dagnew Z, Tafere Y, Bereka TM, Bitewa YB. Time to death among HIV-infected under-five children after initiation of anti-retroviral therapy and its predictors in Oromiya liyu zone, Amhara region, Ethiopia: a retrospective cohort study. BMC Pediatrics. 2022;22(1):5. doi: 10.1186/s12887-021-03072-6 34980032 PMC8722209

[8] Ministry of Health. Consolidated guidelines for the prevention and treatment of HIV and aids in Uganda; 2020 [cited 2022 Jul 25]. Available from: https://dsduganda.com/wp-content/uploads/2023/05/Consolidated-HIV-and-AIDS-Guidelines-20230516.pdf

[9] Kiyaga C, Narayan V, McConnell I, Elyanu P, Kisaakye LN, Joseph E, et al. Uganda’s “EID Systems Strengthening” model produces significant gains in testing, linkage, and retention of HIV-exposed and infected infants: an impact evaluation. PLoS One. 2021;16(2):e0246546. doi: 10.1371/journal.pone.0246546 33539425 PMC7861549

[10] Ministry of Health. Consolidated guidelines for the prevention and treatment of HIV in Uganda; 2018 [cited 2023 Sep 7]. Available from: https://platform.who.int/docs/default-source/mca-documents/policy-documents/guideline/UGA-RH-43-01-GUIDELINE-2018-eng-Final-Uganda-HIV-Guidelines.pdf

[11] Kiyaga C, Lee HH, Allain J-P. Adherence to early infant diagnosis testing algorithm, a challenge to early infant diagnosis program in resource limited settings of Uganda. J HIV Clin Sci Res. 2015;2(2):030–9.

[12] Essajee S, Bhairavabhotla R, Penazzato M, Kiragu K, Jani I, Carmona S, et al. Scale-up of early infant HIV diagnosis and improving access to pediatric HIV care in global plan countries: past and future perspectives. J Acquir Immune Defic Syndr. 2017;75 Suppl 1:S51–8. doi: 10.1097/QAI.0000000000001319 28398997

[13] Namukwaya Z, Barlow-Mosha L, Mudiope P, Kekitiinwa A, Matovu JN, Musingye E, et al. Use of peers, community lay persons and Village Health Team (VHT) members improves six-week postnatal clinic (PNC) follow-up and Early Infant HIV Diagnosis (EID) in urban and rural health units in Uganda: a one-year implementation study. BMC Health Serv Res. 2015;15:555. doi: 10.1186/s12913-015-1213-5 26666331 PMC4678627

[14] Kiyaga C, Sendagire H, Joseph E, McConnell I, Grosz J, Narayan V, et al. Uganda’s new national laboratory sample transport system: a successful model for improving access to diagnostic services for early infant HIV diagnosis and other programs. PLoS One. 2013;8(11):e78609. doi: 10.1371/journal.pone.0078609 24236026 PMC3827263

[15] PEPFAR. Uganda country operational plan COP21 strategic direction summary; 2021 [cited 2023 Sep 6]. Available from: https://www.state.gov/wp-content/uploads/2021/09/Uganda_SDS_Final-Public_Aug-13-2021.pdf

[16] Ministry of Health. Evaluation of the adoption, implementation and effectiveness of PMTCT retention interventions in Uganda: 2015 – 2020. 2022.

[17] Ministry of Health. Consolidated guidelines for the prevention and treatment of hiv and aids in Uganda; 2020 [cited 2022 Aug 6]. Available from: https://differentiatedservicedelivery.org/Portals/0/adam/Content/HvpzRP5yUUSdpCe2m0KMdQ/File/Uganda_Consolidated%20HIV%20and%20AIDS%20Guidelines%202020%20June%2030th.pdf

[18] Ministry of Health. Impact of the national program for prevention of mother - to child transmission of HIV in Uganda, evaluation report 2017 – 2019; 2022 [cited 2023 Dec 11]. Available from: https://www.health.go.ug/wp-content/uploads/2022/08/PMTCT-E-REPORT_.pdf

[19] STRIVE. Measuring HIV stigma and discrimination; 2018 [cited 2025 Feb 25]. Available from: https://strive.lshtm.ac.uk/system/files/attachments/STRIVE%20stigma%20measurement.pdf?utm_source=chatgpt.com

[20] Abuse NIoA, Alcoholism. The physicians’ guide to helping patients with alcohol problems. US Department of Health and Human Services, Public Health Service, National …; 1995.

[21] George A, Stead TS, Ganti L. What’s the risk: differentiating risk ratios, odds ratios, and hazard ratios? Cureus. 2020;12(8). 10.7759/cureus.10047 PMC7515812 32983737

[22] Robbins AS, Chao SY, Fonseca VP. What’s the relative risk? A method to directly estimate risk ratios in cohort studies of common outcomes. Ann Epidemiol. 2002;12(7):452–4. doi: 10.1016/s1047-2797(01)00278-2 12377421

[23] Knol MJ, Algra A, Groenwold RHH. How to deal with measures of association: a short guide for the clinician. Cerebrovasc Dis. 2012;33(2):98–103. doi: 10.1159/000334180 22156574

[24] Kamble S, Gawde N, Goel N, Thorwat M, Nikhare K, Bembalkar S, et al. Access, timeliness and retention for HIV testing under early infant diagnosis (EID) program, India. Sci Rep. 2023;13(1):5638. doi: 10.1038/s41598-023-32056-y 37024531 PMC10078073

[25] Gawde N, Kamble S, Goel N, Nikhare K, Bembalkar S, Thorwat M, et al. Loss to follow-up of HIV-exposed infants for confirmatory HIV test under Early Infant Diagnosis program in India: analysis of national-level data from reference laboratories. BMC Pediatr. 2022;22(1):602. doi: 10.1186/s12887-022-03656-w 36253771 PMC9578277

[26] Webb K, Chitiyo V, Mahachi N, Huruva Mukungunugwa S, Mushavi A, Zizhou S, et al. Brief report: improving early infant diagnosis observations: estimates of timely HIV testing and mortality among HIV-exposed infants. J Acquir Immune Defic Syndr. 2020;83(3):235–9. doi: 10.1097/QAI.0000000000002263 31913988 PMC7012331

[27] Hassan AS, Sakwa EM, Nabwera HM, Taegtmeyer MM, Kimutai RM, Sanders EJ, et al. Dynamics and constraints of early infant diagnosis of HIV infection in Rural Kenya. AIDS Behav. 2012;16(1):5–12. doi: 10.1007/s10461-010-9877-7 21213034 PMC3254874

[28] Finocchario-Kessler S, Brown M, Maloba M, Nazir N, Wexler C, Goggin K, et al. A pilot study to evaluate the impact of the HIV Infant Tracking System (HITSystem 2.0) on Priority Prevention of Mother-to-Child Transmission (PMTCT) outcomes. AIDS Behav. 2021;25(8):2419–29. doi: 10.1007/s10461-021-03204-0 33709212 PMC8224224

[29] Vrazo AC, Firth J, Amzel A, Sedillo R, Ryan J, Phelps BR. Interventions to significantly improve service uptake and retention of HIV-positive pregnant women and HIV-exposed infants along the prevention of mother-to-child transmission continuum of care: systematic review. Trop Med Int Health. 2018;23(2):136–48. doi: 10.1111/tmi.13014 29164754

[30] Takada S, Weiser SD, Kumbakumba E, Muzoora C, Martin JN, Hunt PW, et al. The dynamic relationship between social support and HIV-related stigma in rural Uganda. Ann Behav Med. 2014;48(1):26–37. doi: 10.1007/s12160-013-9576-5 24500077 PMC4104218

[31] Turan B, Budhwani H, Fazeli PL, Browning WR, Raper JL, Mugavero MJ, et al. How does stigma affect people living with HIV? The mediating roles of internalized and anticipated HIV stigma in the effects of perceived community stigma on health and psychosocial outcomes. AIDS Behav. 2017;21(1):283–91. doi: 10.1007/s10461-016-1451-5 27272742 PMC5143223

[32] Blake Helms C, Turan JM, Atkins G, Kempf M-C, Clay OJ, Raper JL, et al. Interpersonal mechanisms contributing to the association between HIV-related internalized stigma and medication adherence. AIDS Behav. 2017;21(1):238–47. doi: 10.1007/s10461-016-1320-2 26864692 PMC4980279

[33] Jones HS, Floyd S, Stangl A, Bond V, Hoddinott G, Pliakas T, et al. Association between HIV stigma and antiretroviral therapy adherence among adults living with HIV: baseline findings from the HPTN 071 (PopART) trial in Zambia and South Africa. Trop Med Int Health. 2020;25(10):1246–60. doi: 10.1111/tmi.13473 32745296 PMC7590062

[34] Sianturi EI, Perwitasari DA, Islam MA, Taxis K. The association between ethnicity, stigma, beliefs about medicines and adherence in people living with HIV in a rural area in Indonesia. BMC Public Health. 2019;19(1):55. doi: 10.1186/s12889-019-6392-2 30634953 PMC6330480

[35] Weiner C. Syncopal attacks and sudden death due to ventricular fibrillation -- a variant of the Jervell or Lange-Nielsen syndrome (author’s transl). Klin Padiatr. 1975;187(5):448–60. doi: 10.1097/qai.0000000000001589 126334

[36] Hatcher AM, Woollett N, Pallitto CC, Mokoatle K, Stöckl H, MacPhail C, et al. Bidirectional links between HIV and intimate partner violence in pregnancy: implications for prevention of mother-to-child transmission. J Int AIDS Soc. 2014;17(1):19233. doi: 10.7448/IAS.17.1.19233 25371218 PMC4220001

[37] Hampanda KM. Intimate partner violence and HIV-positive women’s non-adherence to antiretroviral medication for the purpose of prevention of mother-to-child transmission in Lusaka, Zambia. Soc Sci Med. 2016;153:123–30. doi: 10.1016/j.socscimed.2016.02.011 26896876 PMC4788551

[38] Yonga AM, Kiss L, Onarheim KH. A systematic review of the effects of intimate partner violence on HIV-positive pregnant women in sub-Saharan Africa. BMC Public Health. 2022;22(1):220. doi: 10.1186/s12889-022-12619-w 35114964 PMC8815228

[39] Hatcher AM, Turan JM, Stöckl H, Woollett N, Garcia-Moreno C, Christofides NJ. Intimate partner violence and HIV treatment adherence in urban South Africa: Mediating role of perinatal common mental disorders. SSM Ment Health. 2022;2:100112. doi: 10.1016/j.ssmmh.2022.100112 36688232 PMC9792377

[40] Schrubbe LA, Stöckl H, Hatcher AM, Calvert C. Sexual violence and antiretroviral adherence among women of reproductive age in African population-based surveys: the moderating role of the perinatal phase. J Int AIDS Soc. 2023;26(6):e26129. doi: 10.1002/jia2.26129 37306126 PMC10258861

[41] Biomndo BC, Bergmann A, Lahmann N, Atwoli L. Intimate partner violence is a barrier to antiretroviral therapy adherence among HIV-positive women: evidence from government facilities in Kenya. PLoS One. 2021;16(4):e0249813. doi: 10.1371/journal.pone.0249813 33882084 PMC8059826

[42] Leddy AM, Weiss E, Yam E, Pulerwitz J. Gender-based violence and engagement in biomedical HIV prevention, care and treatment: a scoping review. BMC Public Health. 2019;19(1):897. doi: 10.1186/s12889-019-7192-4 31286914 PMC6615289

[43] United Nations. Uganda: violence against women unabated despite laws and policies; 2023 [cited 2023 Sep 19]. Available from: https://www.un.org/africarenewal/news/uganda-violence-against-women-unabated-despite-laws-and-policies

[44] Abebe AM, Kassaw MW, Shewangashaw NE. Level of knowledge about prevention of mother-to-child transmission of HIV option B and associated factors among ANC clients in Kombolcha Town, South Wollo Amhara Regional State, Ethiopia, 2017. HIV/AIDS (Auckland, NZ). 2020;12:79. 10.2147/HIV.S242166 PMC7037140 32110115

[45] Zacharius KM, Basinda N, Marwa K, Mtui EH, Kalolo A, Kapesa A. Low adherence to Option B+ antiretroviral therapy among pregnant women and lactating mothers in eastern Tanzania. PLoS One. 2019;14(2):e0212587. doi: 10.1371/journal.pone.0212587 30794633 PMC6386496

