# Supplementary material for: Complete testing coverage for the early infant diagnosis algorithm and associated factors among infants exposed to HIV, Uganda, 2017–2019
Source: PLoS One. 2025 Jun 10;20(6):e0324338. doi: 10.1371/journal.pone.0324338 (PMC12151357; doi:10.1371/journal.pone.0324338)
Supplement: S1 Table — (DOCX) [file pone.0324338.s001.docx]

**S1 Table**. **Sensitivity analysis for factors associated with complete testing coverage according to the early infant diagnosis algorithm among Infants exposed to HIV, Uganda, 2017-2019**

| **Variable** | Main analysis | Missing=CTC | Missing=Non-CTC |
| --- | --- | --- | --- |
| aRR (95% CI) | | | |
| Number of pregnancies, mean(±SD) | 1.03 (1.01-1.46) | 1.03(1.01-1.45) | 1.03 (1.02-1.49) |
|  |  |  |  |
| Reported experience of sexual violence | 0.82 (0.73-0.93) | (0.83-0.94) | 0.82 (0.72-0.92) |
|  |  |  |  |
| Experienced discrimination | 0.77(0.65-0.92) | 0 .76 (0.65-0.91) | 0 .78 (0.66-0.93) |

Results are presented as adjusted risk ratios (aRR) with 95% confidence intervals (CI). The main analysis excludes infants with missing data, while sensitivity analyses assume all missing cases were either complete testing coverage (Missing = CTC) or non-complete testing coverage (Missing = Non-CTC). Only statistically significant findings are presented
